# Transcatheter Therapy for the Tricuspid Valve: A Focused Review of Edge-to-Edge Repair and Orthotopic Valve Replacement

**DOI:** 10.1007/s11886-024-02051-4

**Published:** 2024-06-17

**Authors:** Mahesh V. Madhavan, Vratika Agarwal, Rebecca T. Hahn

**Affiliations:** 1 NewYork-Presbyterian Hospital, Columbia University Irving Medical Center, 177 Fort Washington Avenue, New York, NY 10032 USA; 2 Cardiovascular Research Foundation, New York, NY USA

**Keywords:** Tricuspid regurgitation, Transcatheter tricuspid valve intervention, Tricuspid transcatheter edge-to-edge repair, Transcatheter tricuspid valve replacement

## Abstract

**Purpose of Review:**

Patients with severe tricuspid regurgitation (TR) are at risk for significant morbidity and mortality. Transcatheter tricuspid valve interventions (TTVI) may offer patients less invasive treatment alternatives to surgery. This review evaluates the most common class of device currently used worldwide to treat TR, tricuspid transcatheter edge-to-edge repair (T-TEER) and orthotopic transcatheter tricuspid valve replacement (TTVR), both of which are now approved in the USA and Europe.

**Recent Findings:**

The first pivotal randomized clinical trial, TRILUMINATE, demonstrated that T-TEER can safely reduce TR and is associated with improved health status outcomes. However, results of this trial have raised questions about whether this device can provide sufficient TR reduction to impact clinical outcomes. Orthotopic TTVR has recently gained attention with initial data suggesting near-complete TR elimination.

**Summary:**

The current review examines the technical features and anatomic limitations of the most commonly used devices for T-TEER and orthotopic TTVR, discusses the current clinical data for these devices, and offers a theoretical construct for device selection.

## Introduction

There has been growing awareness regarding the prevalence and impact of tricuspid regurgitation (TR) on outcomes [1•]. Clinically significant TR is highly prevalent, afflicting nearly 5 million individuals in the United States and Europe with increasing prevalence in patients of advanced age and women and is associated with substantial morbidity and mortality [1–5]. There are however no Class I medical therapy recommendations to treat symptomatic severe TR in the current guidelines given the paucity of evidence in this understudied population [6, 7]. To add to the management challenges, the only Class I indication for surgical therapy in the American guidelines is in the setting of correction of concomitant left-sided valve surgery [6] with isolated tricuspid valve (TV) surgery associated with high morbidity and mortality [8, 9]. The poor outcomes associated with isolated TV surgery is in large part due to the late presentation of these patients, related to a number of factors: (1) lack of guideline recommendations and limited validation for risk assessment scores [10], (2) underappreciation of the independent association of TR with outcomes particularly in the setting of secondary disease [11], (3) non-specific symptoms preventing early clinical diagnosis [10], and (4) underutilization of quantitative imaging modalities for both TR and right ventricular (RV) assessment [12, 13].

These challenges form the justification for the rapid growth of transcatheter tricuspid valve interventions (TTVI) currently under investigation [14]. These fall into 4 broad categories based on their primary mechanism of action: leaflet approximation, annular reduction, orthotopic valve replacement, and heterotopic valve replacement. Both a tricuspid transcatheter edge-to-edge repair (T-TEER) and a transcatheter tricuspid valve replacement (TTVR) systems have been recently approved in the USA, and devices belonging to all four categories have now been approved for use in Europe, although annular reduction and heterotopic valve replacement devices have not seen broad adoption. The majority TTVI implants have been leaflet approximation and orthotopic valve replacement devices. The current review will examine the technical features and anatomic limitations of the most commonly used devices for T-TEER and orthotopic TTVR, discuss the current clinical data related to patient selection and outcomes, and offer a theoretical construct for device choice.

## Current Device Technology

### Tricuspid Transcatheter Edge-to-Edge Repair

Tricuspid-specific TEER is currently the most used TTVI treatment strategy across the world. The T-TEER devices aim to reproduce the Alfieri surgical technique by facilitating improved leaflet approximation to reduce valvular regurgitation [15]. These devices grasp and bring together opposing leaflets, thereby reducing coaptation gaps and severity of TR. Of the T-TEER devices, TriClip^TM^ (Abbott Structural Heart, Santa Clara, CA) and PASCAL (Edwards Lifesciences, Irvine, CA) are the most extensively studied. TEER in the tricuspid position is typically performed under general anesthesia with two-dimensional (2D) and three-dimensional (3D) transesophageal echocardiogram (TEE) guidance which has challenges given the far-field imaging of a large valve with thin leaflets [12, 16]. The recent commercial availability of 3D intracardiac echocardiography has provided an adjunctive imaging tool for this procedure [17]. While T-TEER has the potential to substantially reduce TR, < 60% of patients will achieve ≤ mild TR [18–20].

The TriClip^TM^ system uses a right heart-specific guide and delivery system and the 4th generation implants: NT and XT clip sizes (4 mm width; 9 [NT] and 12 [XT] mm arm length), a wider implant size of 6 mm is available with both arm lengths (6 mm width; 9 [NTW] and 12 [XTW] mm arm length). The two rigid arms of cobalt-chromium alloy have flexible nitinol-based “grippers” with longitudinally arranged frictional elements, four for the NT and NTW and six for the XT and XTW. There is independent and controlled gripper action and an active locking mechanism. There are two working catheters for positioning the device, the clip delivery system (CDS), and the guide catheter; for optimal steerability, the two markers of the CDS must “straddle” the tip of the guide.

The PASCAL system for either mitral or tricuspid TEER has three working catheters with the device attached to the distal end of the inner implant catheter. With this design, there is high range of motion and maneuverability without dictating the relative positions of the catheters. The device itself is nitinol throughout with two spring-loaded, curved paddles (10 mm wide for P10 and 6 mm wide for ACE) with horizontally arranged retention elements along the distal end of the paddles and a central spacer of varying diameters (smaller for the ACE). The nitinol clasps can be controlled individually, enabling either simultaneous or independent leaflet capture and with passive closing mechanism (based on nitinol shape memory).

### Transcatheter Tricuspid Valve Replacement

Orthotopic TTVR has the potential to completely eliminate TR, with anatomic feasibility dependent on the anchoring mechanism of the particular device. Potential mechanisms for maintenance of device stability include the use of radial force against the annular anatomy, tricuspid leaflet/annular engagement, and non-TV anchoring mechanisms such as the septum or vena cava [21].

The EVOQUE tricuspid valve replacement system (Edwards Lifesciences, Irvine, CA) is the most extensively studied of these devices [22–26]. The device is comprised of a 27-mm trileaflet bovine pericardial tissue valve implant in a nitinol frame with diameters of 44 mm, 48 mm, and 52 mm. Nine anchors are attached to the outer frame for implantation stability, with a sealing skirt to minimize paravalvular leak. The delivery system is inserted over an echocardiographically positioned guidewire and advanced across the TV. After position and trajectory are confirmed, the nine anchors are exposed by retracting the capsule to ensure that anchors remain below the leaflet tips and above the papillary muscle heads. During further expansion, anchor tips are positioned below the annulus ensuring leaflets are captured. At each stage of the procedure, all nine anchors must be individually imaged for correct positioning [27]. After optimal anchor positioning and confirmed leaflet capture, the EVOQUE valve is fully deployed and released from the delivery system. TEE-guided positioning this TTVR utilizes advanced 3D imaging capabilities and is frequently less challenging than the T-TEER devices.

## Anatomic Suitability

**Fig. 1:**
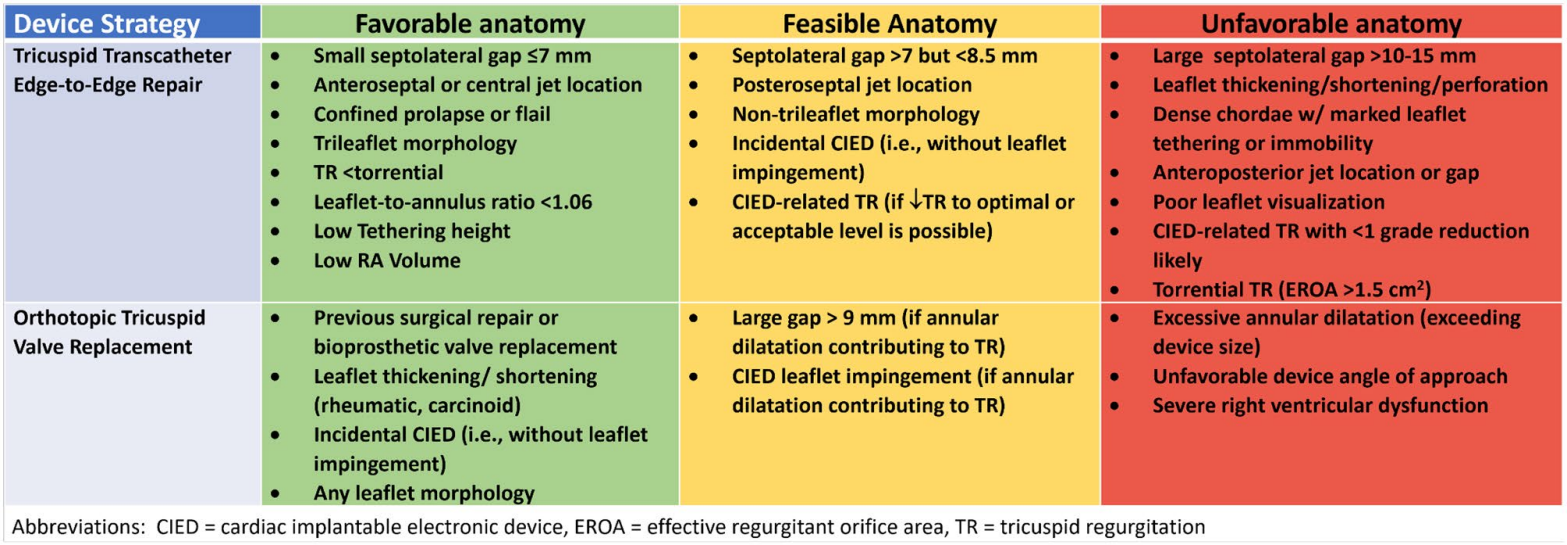
Anatomic suitability of tricuspid transcatheter edge-to-edge repair and orthotopic tricuspid valve replacement

The Tricuspid Valve Academic Research Consortium (TVARC) [28] defines that the adequate performance of a transcatheter device whose purpose is a reduction in TR should include the absence of tricuspid stenosis (TV area ≥ 1.5 cm^2 ^or TV area index ≥ 0.9 cm^2^ /m^2^ [≥ 0.75 if BMI > 30], Doppler index < 2.2, mean gradient < 5 mmHg) and reduction of total TR to optimal (≤ mild [1+]) or acceptable (≤ moderate [2+]). Multiple studies have shown that worse outcomes are associated with greater severity of residual TR [18, 29–31]. Thus, anatomic parameters which may predict procedural success may be used to identify suitable candidates for these procedures and are shown in Fig. 1.

## T-TEER

In most T-TEER studies, procedural success is defined as a TR reduction to ≤ moderate or 2+. In this setting, predictors of success have been identified in various small studies and include the following: location of jet, size of the coaptation gap, leaflet morphology (i.e., number of leaflets [32]), leaflet thickness or calcification, leaflet mobility, complexity of subvalvular apparatus, severity of TR, leaflet-to-annulus ratio, location and extent of CIED-related TR, tethering height, and right atrial volume (Fig. 1) [30, 33–40]. Very large coaptation gaps, torrential TR, markedly thickened or immobile leaflets, and CIED-related TR where the device is adherent to leaflets or subvalvular apparatus precluding adequate TR reduction may be relative exclusion for this technology. In addition, visualization of the leaflets during the procedure is required and the use of TEE with or without adjunctive intracardiac echocardiographic imaging, heavily relying on 3D functions, is also an “anatomic” requirement.

## TTVR

The anatomic requirements for TTVR are primarily related to the ability to position the device within the annular plane and the anchoring mechanism. Thus, different devices will have different anatomic restrictions. A balloon-expandable valve implantation within a surgical valve prosthesis or ring was the first type of TTVR performed [41–43]. However TTVR for native TV disease has progressed rapidly, moving from a transatrial approach [44–46] to transfemoral [23, 24, 47] or transjugular venous approaches [48]. Unlike the T-TEER devices, large coaptation gaps, torrential TR, complex leaflet morphologies, markedly thickened or immobile leaflets, and CIED-related TR are not typically exclusions to TTVR. For these devices, significant determinants of feasibility are the size of the current devices as well as the ability to steer the device to obtain a coaxial implantation trajectory, in large part determined by the size of the implant device and available right heart space. Although also described for T-TEER [49], acute afterload mismatch and RV failure following obliteration of severe TR may theoretically be a greater concern for TTVR since ≤ mild TR may be achieved in > 90% of patients [26, 50••].

## Review of Device Outcomes

Outcomes for the T-TEER and TTVR are limited to early feasibility studies, and post-market registries with only one randomized controlled trial (RCT) currently reported. To understand difference in efficacy and outcomes of these trials, it is important to know the baseline characteristics of the trials (Table 1) and the reported procedural outcomes (Table 2).

**Table 1:** Comparison of baseline clinical characteristics in bRIGHT, TRILUMINATE, and TRISCEND II

**Baseline characteristics**	**bRIGHT** **T-TEER Group** [30] **(*N* = 511)**	**TRILUMINATE** **T-TEER Group** [19••] **(*N* = 175)**	**TRILUMINATE** **Control Group** [19••] **(*N* = 175)**	**TRISCEND II** **TTVR Group** [50••] **(*N* = 96)**	**TRISCEND II** **Control Group** [50••] **(*N* = 54)**
**Age (years)**	78.9 ± 7.1	78.0 ± 7.4	77.8 ± 7.2	79.4 ± 7.7	78.2 ± 8.3
**Female sex**	56.0	56.0	53.7	82.3	75.9
**NYHA Class III/ IV**	80.0	59.4	55.4	79.2	75.9
**Hypertension**	86.7	81.1	80.6	91.7	87.0
**Diabetes mellitus**	22.3	16.0	15.4	-	-
**Atrial fibrillation**	86.3	87.4	92.6	97.9	96.3
**Prior stroke**	8.0	6.3	10.9	19.8	5.6
**Renal dysfunction**	39.5	35.4	35.4	50.0	57.4
**COPD**	13.1	10.9	13.7	19.8	16.7
**Peripheral vascular disease**	11.0	9.1	10.3	-	-
**Prior CABG**	11.5	17.7	20.6	10.4	24.1
**LVEF**	55.8 ± 10.6	59.3 ± 9.3	58.7 ± 10.5	55.1 ± 8.6	52.4 ± 11.6
**Right ventricular function**	RV FAC: 39.4 ± 8.4 TAPSE: 1.7 ± 0.44	RV FAC: 36.6 ± 5.5 TAPSE > 1.7 cm: 48%	RV FAC: 37.2 ± 6.3 TAPSE > 1.7 cm: 41.2%	-	-
**Prior aortic intervention**	9.2	15.4	15.4	-	-
**Prior mitral intervention**	26.8	Surgical mitral valve repair: 8.0 Percutaneous mitral valve repair: 10.3 Mitral valve replacement: 5.7	Surgical mitral valve repair: 5.1 Percutaneous mitral valve repair: 12.6 Mitral valve replacement: 5.1	-	-
**Functional TR**	90.0	94.8	92.9	77.4*	70.4*
**Baseline TR Severity**					
**Moderate**	2.0	2.3	1.2	-	-
**Severe**	10.0	25.4	29.7	43.8	40.7
**Massive**	61.3	21.4	18.2	21.9	27.8
**Torrential**	26.7	50.9	50.9	34.4	31.5
**Permanent Pacemaker/ICD**	22.5	16.0	13.7	36.5	42.6
**KCCQ score**	44.5 ± 22.6	56.0 ± 23.4	54.1 ± 24.2	49.1 ± 21.5	49.7 ± 22.3
**HFH within 1 year**	40.3	25.1	25.1	31.3	31.5

Data are presented as mean ± SD or %

*CABG* coronary artery bypass graft surgery, *COPD* chronic obstructive pulmonary disease, *HFH* heart failure hospitalization, *ICD* implantable cardiac defibrillator, *KCCQ* Kansas City Cardiomyopathy Questionnaire, *LVEF* left ventricular ejection fraction, *NYHA* New York Heart Association, *RV FAC* right ventricular fractional area change (%), *TAPSE* tricuspid annular plane systolic excursion, *TR* tricuspid regurgitation, *T-TEER* tricuspid transcatheter edge-to-edge repair

^*^8.3–16.7% with indeterminate or mixed TR etiology

**Table 2:** Complications reported in the literature for tricuspid transcatheter edge-to-edge repair (T-TEER) and transcatheter tricuspid valve replacement (TTVR) devices

**Category**		**Complication (within 30d unless indicated)**	**T-TEER rate (references)**	**TTVR rate (references)**
Device-related	Structural failure	Single leaflet device attachment	2.6–7.0% [18, 19, 30, 31, 52, 55] 4.6-7.7% (at 1 yr) [51, 56]	NA
		Device embolization	0% [19, 30, 52]	0-2.6% [23, 25, 26, 65]
		Device thrombus	0% [19, 30]	4% [23] 32% (at 2yrs) [65]
		Reintervention	0–1.8% [19, 30, 52, 55]	0–2.6% [23, 25, 26, 65] 4.0% (at 1 yr) [26]
	Functional impairment	Residual TR > 2+	12–48% [19, 30, 31, 52, 55, 56]	0–4% [23, 25, 26] 1.2% (at 6 mo) [50••] 0% (at 1 yr) [26]
		Transtricuspid gradient > 5 mmHg or TS	3–9% [19, 31, 52]	5.3% [65]
	Other	Pacemaker implant	0-2.9% [19, 30]	8–14.7% [23, 25, 26, 50, 65]
Procedure-related	Access-site vascular	Major vascular complications	0–3.1% [31, 55, 56]	2.3% [25, 26] 2.7% (at 1 yr) [26]
	Cardiac structural damage	Pericardial effusion/tamponade	0% [19••]	NR
		RV perforation	NR	NR
	Bleeding	Major bleeding	5.9–7.7% [30, 55, 56] 5.2-9.2% (at 1 yr) [19, 56]	10.5–16.9% [23, 25, 26, 50 25.5% (at 1 yr) [26]
	Thromboembolic	Myocardial infarction	0% [30, 51, 55] 0-1.2% (at 1 year) [51, 56]	0-1.1% [23, 25, 26, 50 0% (at 1 yr) [26]
		Stroke	0.4-1.5 [30, 55, 56] 1.2-4.6% [19, 51, 56]	0-0.6% [23, 25, 26, 50 1.3% (at 1 yr) [26]
Cardiovascular mortality			0–3.1% [19, 30, 55, 56] 4; 4.8-7.7% (at 1 yr) [19, 51, 56]	1.7-3.2% [25, 26, 50 9.4% (at 1 yr) [26]

*BARC* bleeding academic research consortium, *NA* not applicable, *NR* not reported, *RV* right ventricular, *SLDA* single leaflet device attachment, *TR* tricuspid regurgitation, *TS* tricuspid stenosis

## T-TEER

The TRILUMINATE early feasibility study demonstrated sustained TR reduction after TriClip^TM^ in an 85-patient study with 2-year follow-up [18, 51, 52]. Echocardiographic markers of right heart size and hemodynamics and quality of life parameters all similarly demonstrated persistent favorable trends at 2 years. Moreover, when compared to baseline data prior to intervention, significant reductions in all-cause hospitalizations were present after T-TEER therapy in individuals with 2-year follow-up (0.66 events per patient year vs. 1.30 events per patient year, *p* < 0.0001).

TriClip^TM^ initially received CE Mark approval in Europe, and the FDA recently approved this device in the USA after reviewing the TRILUMINATE Pivotal RCT results [19••]. This study randomized 350 patients of intermediate or greater surgical risk patients with severe TR to T-TEER with medical therapy versus medical therapy alone in 1:1 fashion. The primary composite endpoint was a hierarchical composite of all-cause death or tricuspid-valve surgery, heart failure hospitalization, and improvement in quality of life measured by KCCQ at 1 year. Baseline patient characteristics of the control and intervention arms of this RCT are presented in Table 1. Mean age of enrolled patients was approximately 78 ± 7 years with approximately 70% presenting with massive or torrential TR. While the primary endpoint was met and favored the T-TEER group (win ratio, 1.48; 95% confidence interval [CI], 1.06–2.13; *p* = 0.02), this was primarily driven by improvements in KCCQ score at 1 year (mean difference, 11.7; 95%CI, 6.8–16.6; *p* < 0.001). Changes in KCCQ scores were significantly associated with degree of residual TR and magnitude of TR reduction at 1 year, and similar improvements in KCCQ were observed across several patient subgroups. No benefits with regards to rates of all-cause death/ TV surgery or heart failure hospitalizations were observed with T-TEER therapy. Reductions in TR severity were noted with TriClip^TM^, as a significantly greater proportion of patients had moderate or less TR at 30 days compared with the medical therapy group (87.0% vs. 4.8%, *p* < 0.001) and similar findings were noted at 1-year follow up (88.9% vs. 5.7%). The majority of patients (98.3%) who underwent T-TEER did not experience major adverse events within 30 days. While procedural success rates were high (87%), major bleeding (5.2%), SLDA (7%), tricuspid mean gradient ≥ 5 mmHg (5%), device embolization (0%), and device thrombosis (0%) were not frequently observed events (Table 2).

A dedicated analysis of health status outcomes delved into further describing the benefits with regards to quality-of-life parameters in the TRILUMINATE trial [53]. Results of this analysis confirmed that health status benefits of T-TEER persisted from 1 month after randomization through 1-year follow-up (mean KCCQ between-group difference 10.4 points, 95% CI 6.3–14.6). Patients who received T-TEER were more likely to be alive and well at 1 year when compared to patients in the medical therapy group (number needed to treat, 3.5). While results were largely consistent across subgroups, patients with preserved cardiac index (≥ 2 L/min/m^2^) appeared more likely to benefit compared to individuals with reduced cardiac index.

More recently, the bRIGHT post-approval study presented further safety and performance data of T-TEER with TriClip^TM^ (Tables 1 and 2) [30]. This prospective, single-arm, open-label, multicenter, post-market registry performed at 26 sites in Europe evaluated outcomes after T-TEER in 511 patients with largely massive or torrential TR (88%) and significant concurrent heart failure symptomatology (80% with New York Heart Association [NYHA] Class III or IV). Successful device implantation was observed in 504 (99%) of patients, and procedural success (implantation success with at least one grade TR reduction noted on discharge or 30 days when appropriate) was achieved in 451 (91%) of patients. After TriClip^TM^ therapy, 80% of patients were noted to have moderate or less TR at on discharge, and these findings were fairly consistent at 30 days. Significant improvements in NYHA functional class, KCCQ scores, and RV echocardiographic parameters were noted at 30 days. The overall adverse event rate at 30 days was 2.7% with a cardiovascular mortality rate noted to be low (0.8%).

The TRILUMINATE Pivotal trial proved that T-TEER with TriClip^TM^ can be a safe and effective treatment for sustained reduction in severe TR (Tables 1 and 2). That the degree of reduction was associated with improvements in KCCQ scores suggests a mechanistic relationship between TR reduction quality-of-life metrics. However, given open label trial and lack of a sham control, it remains unclear if the perceived benefits in KCCQ improvement may have been, at least in part, due to unblinded nature of the study. Moreover, lack of benefits with regards to all-cause death or need for TV surgery, heart failure hospitalizations, or 6-min walk test were not encouraging. Questions remain regarding whether outcomes of TRILUMINATE may have been impacted by patient selection or duration of follow-up after intervention. Specifically with regards to patient selection, key differences between the TRILUMINATE RCT and bRIGHT have been noted. In addition to observed differences in baseline characteristics, patients in bRIGHT more frequently had massive and torrential TR as well as trends towards higher proportion of New York Heart Association (NYHA) III/IV symptoms, lower KCCQ scores, and more frequently were admitted with heart failure hospitalizations in the year prior to enrollment [19, 30]. Thus, it remains to be determined whether specifically patient subsets (e.g., potentially higher risk and more symptomatic patients) may derive more benefit from T-TEER therapy.

After description of the initial PASCAL compassionate use experience in high surgical risk or inoperable patients [54], results of the CLASP TR Early Feasibility Study up to 1 year after treatment have been made available [55, 56]. The recently presented 1-year report summarizes outcomes after T-TEER with PASCAL in a cohort of 65 patients [56]. Significant reductions in TR severity and improvements in NYHA functional class, KCCQ score, and 6-min walk test were observed at 30 days, and the initial 30-day benefits with regards to these parameters remained consistent at 1-year follow-up. Rates of major adverse events were 9.2% at 30 days and 16.9% at 1 year, driven mainly by cardiovascular mortality and severe bleeding events. Only three (4.6%) SLDA events were observed in this study (Table 2).

Given narrower profile and longer clasps, some have suggested that the PASCAL Ace device design may prove to be beneficial for use in the tricuspid space in the presence of anatomical characteristics such as dense chordae, annular shape and size, and wide coaptation gaps [57]. The recent report from the PASTE multicenter registry (PASCAL for Tricuspid Regurgitation—A European Registry) studied 235 high-risk patients, most with ≥ severe, functional TR, with after commercial approval in Europe provided more insights [31]. Overall procedural success was 78%, and sustained reduction in TR or ≤ moderate TR in 78% of patients at furthest follow-up available (~ 6 months). This analysis suggested that treatment with both the PASCAL and PASCAL Ace device may result in similar results, resulting in comparable reduction in TR to moderate or less by echocardiographic core laboratory analysis.

The currently underway, pivotal, CLASP II TR RCT (NCT04097145) which aims to randomize 870 patients in 2:1 fashion between treatment with PASCAL T-TEER and guideline-directed medical therapy will certainly provide more insights regarding TTVI treatment of severe, symptomatic TR. The primary endpoint of this study is all-cause mortality, RV assist-device implantation or heart transplant, TV intervention, heart failure hospitalizations, and quality of life improvement at 24 months of follow up. Data from the roll-in cohort of 73 patients appears to be promising, with significant improvements in TR severity, NYHA class, KCCQ score, and RV remodeling and function noted in this non-randomized patient cohort [58].

## TTVR

Several TTVR devices are under development including the following: LuX-Valve (Jenscare biotechnology Co. Ningbo, China), Trisol (Trisol Medical, Yokneam, Israel), CardioValve (CardioValve Ltd., Yehuda, Israel), VDyne (VDyne Inc. Maple Grove, Minnesota), and Topaz (Tricares, Aschheim, Germany). Currently, the largest number of TTVR implants have been the EVOQUE tricuspid valve replacement system.

First-in-human experience with the EVOQUE system in 27 patients presented with follow-up data available up 1 year after TTVR [23, 24]. At baseline, all patients had ≥ severe TR with 89% experiencing NYHA class III or IV symptoms. Significant reductions in TR severity and improvements in NYHA functional class were noted at 30 days. By 1 year, 96% of patients had ≤ moderate TR, and sustained improvements in NYHA functional class were noted. The overall mortality rate at 1 year was 7%.

Subsequently, the prospective, single-arm, multicenter TRISCEND (Edwards EVOQUE Tricuspid Valve Replacement: Investigation of Safety and Clinical Efficacy after Replacement of Tricuspid Valve with Transcatheter Device) studied outcomes in 56 patients after TTVR (Tables 1 and 2) [25]. At baseline, 91% of patients had ≥ severe TR. Thirty-day outcomes demonstrated reduction in TR to mild or less in 98% of patients. Composite major adverse event rate at 30 days was 26.8%, due to 1 cardiovascular death after a failed intervention, 2 reinterventions for device embolization, 1 major access site or vascular complication, and 15 non-fatal bleeding events. Significant improvements in NYHA functional class, KCCQ scores, and 6-min walk tests were also noted at 30 days. A larger analysis of 176 patients with 1-year follow-up demonstrated high rates of device success (94.4%) with 97.6% of patients having no to mild TR at 1-year follow-up [26]. Low rates of cardiovascular mortality (9.4%) were noted at 1 year, and the Kaplan–Meier estimate for heart failure hospitalization was 11.6 ± 2.6%. High rates of severe bleeding (25.5%) and pacemaker requirement were observed (13.3%) at 1 year.

The pivotal TRISCEND II RCT (NCT04482062) randomized 400 patients to the EVOQUE TTVR system versus optimal medical therapy for severe TR. Results of the first 150 patients who were randomized and treated were presented at the 2023 Transcatheter Cardiovascular Therapeutics conference [50••]. Baseline clinical characteristics of these patients are presented in Table 3. At enrollment, > 50% of patients had greater than severe or massive TR. Initial findings demonstrated a 77.1% reduction in TR at 6-month follow-up in patients randomized to the EVOQUE TTVR system (*N* = 96) when compared with medical therapy (*N* = 37) (*p* < 0.001). A substantial difference in KCCQ scores (*Δ* = 17.8) was also noted between the TTVR and medical therapy groups at 6 months. Trends in NYHA functional class and 6-min walk test also seemed to favor TTVR. Notably, this study also lacked a sham control in the medical therapy group. These results led to recent FDA approval of the EVOQUE TTVR system. Complete 1-year results of the total 400 patient cohort, including further clinical and echocardiographic outcomes, are forthcoming.

**Table 3:** Summary of procedural and device-related strengths and weaknesses

**T-TEER**	**TTVR**
**Strengths**	**Strengths**
• Feasible (with caveats) for a variety of TV etiologies • Low thrombotic risk • Low likelihood of CIED jailing or dysfunction • Low likelihood of new conduction defects • Theoretically lower risk of acute RV afterload mismatch	• Appropriate for broad range of TV etiologies (including CIED-related TR) • Appropriate for broad range of leaflet morphologies • Appropriate for large coaptation gaps • Appropriate for torrential TR • Low rate of residual TR > 2+ (~ 0–4%)
**Weaknesses**	**Weaknesses**
• Single leaflet device attachment (~ 2.6–7%) • Residual TR > 2+ (~ 12–48%) • Demanding procedural imaging	• Device thrombosis (up to 32% over 2 yr follow-up) • New pacemaker requirement (~8–15%) • Major bleeding (~11–17%)

## Algorithm for Device Choice

**Fig. 2:**
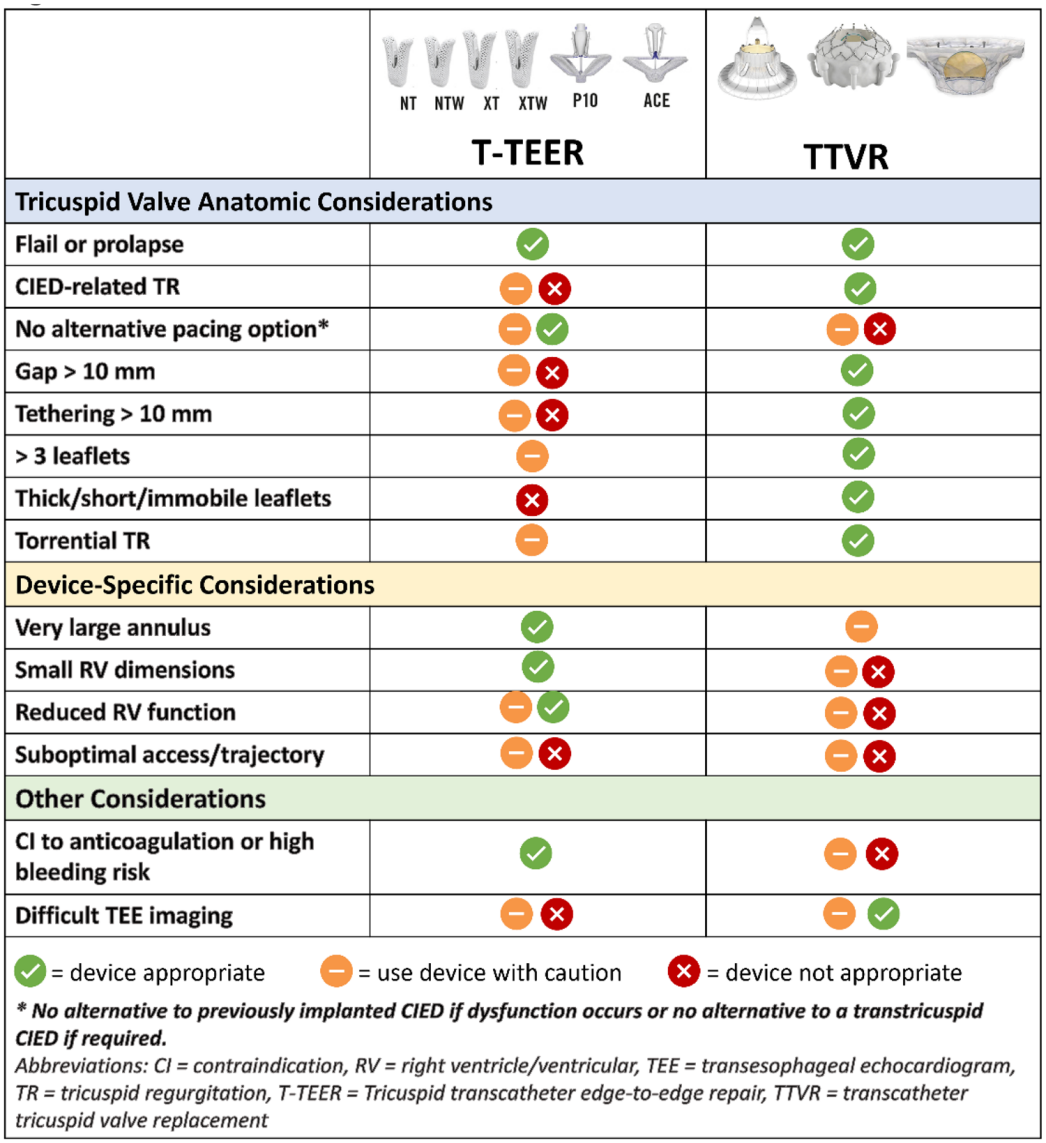
Parameters to consider device choice

Following medical optimization and imaging assessment with transthoracic echocardiogram and TEE for severe symptomatic TR, the structural heart valve team must consider a number of anatomic and clinical factors when determining optimal device selection for an individual patient (Tables 3, Fig. 2).

### Etiology of TR

The new etiologic classification divides TR into primary diseases of the leaflets, secondary diseases (with normal leaflets), and CIED-related TR. Secondary disease is further divided into atrial secondary disease with annular and atrial dilatation being the main driver of leaflet malcoaptation and ventricular secondary disease with ventricular dilatation and leaflet tethering resulting in malcoaptation. These etiologies may determine the appropriateness of each class of TTVI.

T-TEER may address primary TR related to degenerative disease but is not appropriate for diseases resulting in leaflet thickening and restriction such as rheumatic disease or carcinoid valvulopathy. TTVR has been used to treat all types of primary disease [23]. Late-stage secondary disease resulting in extreme tethering or low leaflet-to-annulus ratios may be more difficult treat with T-TEER (see discussion of coaptation gaps below); for these patients, TTVR may be effective in reducing TR to ideal levels. Orthotopic TTVR devices are primarily limited by the large annular dimensions which exceed available device sizes. Efforts are underway to develop larger TTVR device sizes for such anatomic circumstances.

CIED-related TR has been recognized as a predictor of TR progression [59, 60]. The diagnosis can typically be made using transthoracic echocardiography with the use of advanced 3D imaging [61]. In the T-TEER trials, up to 23% of patients have a prior pacemaker; however, in the TTVR trials, up to 43% of patients have a prior pacemaker **(**Table 1). This difference is likely related to the feasibility and efficacy of T-TEER in the setting of CIED-related TR. T-TEER may be feasible even in the setting of CIED-related disease, if there is TR seen distant to the interaction. TTVR can be implanted whether there is CIED-related or CIED-incidental TR. However, the risks for lead interaction or dysfunction with TTVR must also be carefully considered. In the setting of a pre-existing CIED lead across the TV annulus, TTVR will result in “jailing” of the lead which may cause CIED dysfunction [43] and result in difficulty with lead extraction. Lead extraction is a management option not only to reduce the TR in CIED-related disease, but also to allow for easier TTVI implantation. Although transvenous lead extraction is relatively safe, finding an alternative pacing strategy must first be determined particularly in pacer-dependent patients [62]. In circumstances where there are no alternative pacing options if CIED dysfunction occurs or a new pacemaker is required after TTVI, or the patient is at high risk for future pacer infection making jailing the lead undesirable, T-TEER may be preferred over TTVR.

### Large Coaptation Gap

Large coaptation gaps have been demonstrated to be a key anatomic predictor of procedural success with T-TEER [34]. Larger coaptation gaps (i.e., > 7 mm) may be associated with greater residual TR and can technically limit optimal placement of T-TEER devices despite availability of longer device arms and independent leaflet capture in both contemporary T-TEER devices as well as the presence of a central spacer with PASCAL. Conversely, large coaptation gaps represent favorable anatomy for orthotopic TTVR, as such a strategy is not dependent on approximating native leaflets.

### Leaflet Tethering

While T-TEER devices can be feasible in the setting of leaflet tethering, significant tethering (> 10 mm) is observed in the setting of advanced RV remodeling and can contribute towards presence of larger coaptation gaps. Therefore, the use of T-TEER in this circumstance is associated with residual TR and poor procedural success. Leaflet tethering typically does not limit consideration to proceed with orthotopic TTVR, assuming annular dimensions are within range for a given device.

### Leaflet Number and Morphology

Non-trileaflet TV anatomy may pose additional challenges to T-TEER therapy, especially in the presence of dense chords, regurgitant jets which extend into commissures, nonuniform leaflet sizes, and limited grasping area which may result in inadequate leaflet grasp. Such anatomic variants may be feasible for T-TEER depending on the additional complexities to the valve apparatus in addition to the non-trileaflet morphology [32]. Abnormal leaflet morphologies including thickened, shortened, or immobile leaflets (e.g., in the setting of carcinoid, endocarditis, or rheumatic heart disease) or leaflet perforation are not favorable anatomies for T-TEER. However, T-TEER may be feasible in primary TR with flail or prolapsed leaflets. Non-trileaflet valves or the abovementioned morphologies generally should not limit the ability to proceed with orthotopic TTVR with the caveat that careful assessment and consideration may be required in the setting of orthotopic TTVR devices which require anchoring on the ventricular aspects of TV leaflets.

### Right Heart Anatomy

The implantation of the devices depends on the ability to achieve coaxiality with landing zone; thus, both approach angle (typically from the vena cava) and the size of the right atrium and RV may affect procedural success. Smaller RV dimensions can limit the ability to maneuver large-bore delivery systems for orthotopic TTVR devices, increasing potential risk for chordal entanglement or RV injury or perforation, and thus depending on RV imaging assessment may range from feasible to unfavorable anatomy for orthotopic TTVR. Given the small profile of the T-TEER device, right heart size is not typically a restriction.

Though afterload mismatch may occur with any TTVI strategy, given that the increase in RV afterload relates directly to the degree of TR reduction, there is greater potential for severe acute increases in afterload with orthotopic TTVR in the setting of near-complete elimination of TR with this strategy.

### Antithrombotic Considerations

Patients with severe TR may be at risk for bleeding due a number of reasons including hepatic or renal dysfunction and coagulopathy. As current practice is to initiate oral anticoagulation after orthotopic TTVR given potential for valve thrombosis in the setting of slower flow and lower pressure in the right heart, patients with a contraindication for anticoagulation may not be optimal candidates for this strategy. It remains to be seen whether alternate antithrombotic strategies, such as short-term anticoagulation followed by antiplatelet therapy, may be an acceptable alternative to long-term anticoagulation after TTVR. Conversely, T-TEER generally does not require anticoagulation in the post-procedural setting and may thus be a more attractive option in patients with risk factors for bleeding.

### TEE Imaging

TEE imaging is required for both T-TEER and TTVR; however, severe challenges with intraprocedural imaging of valve leaflets and subvalvular anatomy are more likely to render T-TEER feasible or potentially even unfavorable depending on the severity of TEE limitations compared when compared with TTVR. The use of advanced 3D imaging (both TEE and intracardiac echocardiography) have reduced imaging limitations of all TTVI procedures.

## Conclusions

Significant advances have been made with regards to transcatheter treatment for valvular heart disease since the inception of the field. There are a number of clinical and anatomic considerations specific to the TR that make catheter-based treatment strategies challenging. T-TEER and TTVR are the most extensively evaluated transcatheter treatments for TR to date. As the field continues to progress, treatment algorithms specific to certain anatomic and physiologic subsets will likely continue to emerge [10, 27, 63, 64]. The recent TRILUMINATE and TRISCEND II pivotal RCTs results prove that T-TEER and TTVR can reduce TR and associated symptoms and highlight some of the strengths and limitations of these two therapies. As these therapies are now FDA approved, it is likely an increasing number of patients will be considered for and treated with these devices. Whether such treatments can improve clinical endpoints such as mortality and heart failure hospitalizations is yet to be determined. Much will be learned from the results of the ongoing and upcoming clinical studies evaluating such therapies (Table 4).

**Table 4:** Summary of completed and ongoing clinical studies for transcatheter tricuspid valve intervention devices

Transcatheter therapy for TR	Authors	Year (if published)	Study design	*N*	Follow-up duration	Primary endpoint	Principle findings (if available)
**T-TEER and leaflet approximation devices**							
**TriClip**^**TM**^							
**TRILUMINATE RCT** [19••]	Sorajja, Hahn, et al	2023	Pivotal RCT	350	1 year	All-cause death (ACD)/tricuspid valve surgery; hospitalization for heart failure (HFH); quality of life (QoL)	• Win ratio for primary endpoint favored T-TEER, driven primarily by improvement in KCCQ score
**bRIGHT** [30]	Lurz et al	2023	Postmarket registry	511	30 days	Acute procedural success	• Acute procedural success in 91% of patients • TR reduced to moderate in majority of patients at 30 days (77%) • Improvements in NYHA functional class and KCCQ scores
**PASCAL**							
**CLASP TR EFS** [55, 56]	Kodali, Hahn, et al	2023	EFS Study	65	1 year	Primary safety and performance outcomes	• Significant reduction in TR sustained to one-year after intervention • All patients achieved at least 1 TR grade reduction • Significant improvements in NYHA functional class, KCCQ score, and 6-min walk test
**PASTE** [31]	Wild et al	2022	Retrospective postmarket registry	235	~ 6 months	Technical and procedural success, echocardiographic and clinical endpoints	• Procedural success in majority of patients (78%) with sustained TR reduction at ~ 6 months • Improvements in NYHA functional class • Similar outcomes between PASCAL and PASCAL Ace device
**CLASP II TR**	Leon, Mack, Davidson, et al	Study underway	Pivotal RCT	870	2 years	ACD, right ventricular assist-device implantation or heart transplant, tricuspid valve intervention, HFH, QoL	Results pending
**Mistral: Matters I/II** [66, 67]	Piayda et al	2023	First-in-human	9	1 year	Safety and performance endpoints	• Reduction in TR grade in all patients • Significant improvement in 6-min walk test and RV fractional change
**FORMA (spacer)** [68, 69]	Asmarats et al	2019	First-in-human	19	3 years	Safety and performance endpoints	• Clinical improvements in NYHA functional class, 6-min walk test, and KCCQ scores • Only moderate reduction in TR with long-term follow-up
**Annuloplasty**							
**Tri-Align: SCOUT I/II** [70, 71]	Meduri, Hahn, et al	2018	EFS Study	39	30 days	Freedom from death with successful access, delivery and retrieval of delivery device system, correct positioning of intended device, and no need for unplanned surgery or re-intervention	• High-rates of technical success at 30-days (82%) but pledget detachment in 5 patients • Improvements in TV annular diameter, TR EROA, and TV area as well as quality of life parameters
**K-Clip** [72]	Zhang et al	2023	First-in-human study	15	30 days	Procedural success: (a) successful delivery and retrieval of the system, (b) correct implantation of at least one device before exiting the cardiac catheterization lab with reduction in post-procedural TR ≥ +1; freedom from surgical or percutaneous intervention before discharge Clinical success: procedural success with any major adverse events at 30 days	• All 15 patients successfully received implants without major adverse cardiovascular events • Improvement in TR by ≥ 2+ and ≥ 3+ grades was 60% and 27%, respectively • Significant improvements in NYHA functional class and KCCQ score were observed
**Cardioband**							
**TriBAND** [73]	Nickenig et al	2021	Postmarket clinical follow-up	61	30 days	Reduction in TR severity between baseline and discharge	• High rates of device success (96.7%) • Majority of patients achieved at least 1 grade reduction in TR (85%)
**Cardioband EFS** [74]	Gray et al	2022	EFS	37	1 year	Echocardiographic, clinical, and quality of life measures	• Majority of patients with at least 2 grade reduction in TR (73%) • Improvements in echocardiographic parameters • Improvements in NYHA functional class and KCCQ score • High rates of severe bleeding (35%)
**Orthotopic TTVR**							
**TRISCEND** [25, 26]	Kodali, Hahn et al	2022	Registry	176	1 year	Safety and performance endpoints	• Reduction to ≤mild TR in 98% of patients; Improvements in KCCQ and 6-min walk test
**TRISCEND II RCT** [50••]	Kodali, Hahn, Lurz, Thourani, et al	Study underway	Pivotal RCT	400	1 year	TR grade reduction and composite including Kansas City Cardiomyopathy Questionnaire (KCCQ), New York Heart Association functional class improvement, 6-min walk test improvement; major adverse events: composite of ACD, right ventricular assist device implantation or heart transplant, tricuspid valve intervention, HFH, KCCQ improvement, NYHA functional class improvement, 6-min walk test improvement	• 77.1% reduction in TR at 6-month follow-up in patients randomized to the EVOQUE TTVR system (*N* = 96) when compared with medical therapy (*N* = 37) [first 150 patients] • Complete trial results pending
**Intrepid EFS**	Latib, Bapat, et al	Study underway	EFS	15	30 days	Rate of implant or delivery related serious adverse events	Results pending
**Heterotopic TTVR**							
**CAVI** [75]	Dreger et al	2020	RCT	28	90 days	Maximal oxygen up-take	• Significant improvement in dyspnea but not maximal oxygen uptake or other secondary endpoints • Cardiac surgery required in 4 patients in CAVI group
**TricValve®—TRICUS EURO** [76]	Estevez-Loureiro et al	2022	CE Mark Study	35	6 months	QoL and NYHA improvement	• High rates of procedural success (94%) with no procedural deaths or conversion to surgery • Significant improvement in NYHA functional class and KCCQ score
**Tricento** [77]	Wild et al	2022	EFS	21	1 year	Technical success	• High rates of technical success (100%) and no in-hospital mortality • Symptomatic improvement in NYHA functional class • Asymptomatic device fracture in 3 patients • Reduced RV EDV in subset of patients

*ACD all-cause death; CAVI* caval valve implantation, *EFS* early feasibility study, *EROA* effective regurgitant orifice area, *HFH* heart failure hospitalization; *KCCQ* Kansas City Cardiomyopathy Questionnaire, *NYHA* New York Heart Association, *QoL* quality of life; *RCT* randomized clinical trial, *RV EDV* right ventricular end-diastolic volume, *TR* tricuspid regurgitation, *T-TEER* tricuspid transcatheter edge-to-edge repair, *TTVR* transcatheter tricuspid valve replacement, *TV* tricuspid valve

## Abbreviations

2D: Two-dimensional
3D: Three-dimensional
CIED: Cardiovascular implantable electronic device
KCCQ: Kansas City Cardiomyopathy Questionnaire
NYHA: New York Heart Association
RCT: Randomized clinical trial
RV: Right ventricle
TEE: Transesophageal echocardiography
TR: Tricuspid regurgitation
T-TEER: Tricuspid transcatheter edge-to-edge repair
TTVI: Transcatheter tricuspid valve intervention
TTVR: Transcatheter tricuspid valve replacement
TV: Tricuspid valve
